# The Relation between Thematic Role Computing and Semantic Relatedness Processing during On-Line Sentence Comprehension

**DOI:** 10.1371/journal.pone.0095834

**Published:** 2014-04-22

**Authors:** Xiaoqing Li, Haiyan Zhao, Yong Lu

**Affiliations:** 1 Key Laboratory of Behavioral Science, Institute of Psychology, Chinese Academy of Sciences, Beijing, China; 2 University of Chinese Academy of Sciences, Beijing, China; 3 Academy of Psychology and Behavior, Tianjin Normal University, Tianjin, China; The National Institutes of Health, United States of America

## Abstract

Sentence comprehension involves timely computing different types of relations between its verbs and noun arguments, such as morphosyntactic, semantic, and thematic relations. Here, we used EEG technique to investigate the potential differences in thematic role computing and lexical-semantic relatedness processing during on-line sentence comprehension, and the interaction between these two types of processes. Mandarin Chinese sentences were used as materials. The basic structure of those sentences is “Noun+Verb+‘le’+a two-character word”, with the Noun being the initial argument. The verb disambiguates the initial argument as an agent or a patient. Meanwhile, the initial argument and the verb are highly or lowly semantically related. The ERPs at the verbs revealed that: relative to the agent condition, the patient condition evoked a larger N400 only when the argument and verb were lowly semantically related; however, relative to the high-relatedness condition, the low-relatedness condition elicited a larger N400 regardless of the thematic relation; although both thematic role variation and semantic relatedness variation elicited N400 effects, the N400 effect elicited by the former was broadly distributed and reached maximum over the frontal electrodes, and the N400 effect elicited by the latter had a posterior distribution. In addition, the brain oscillations results showed that, although thematic role variation (patient vs. agent) induced power decreases around the beta frequency band (15–30 Hz), semantic relatedness variation (low-relatedness vs. high-relatedness) induced power increases in the theta frequency band (4–7 Hz). These results suggested that, in the sentence context, thematic role computing is modulated by the semantic relatedness between the verb and its argument; semantic relatedness processing, however, is in some degree independent from the thematic relations. Moreover, our results indicated that, during on-line sentence comprehension, thematic role computing and semantic relatedness processing are mediated by distinct neural systems.

## Introduction

Sentence comprehension involves timely computing different types of relations, which include the morphosyntactic, semantic, and thematic relations between its verbs and arguments. In the past years, lots of studies have focused on the interaction between semantic processing and syntactic processing [1]–[3]. Thematic processing is also an important aspect of sentence comprehension. It involves the assigning thematic roles to sentence arguments (i.e., whether an argument is the actor or the undergoer of the event being described). This study aimed to examine the interaction between thematic relation processing and lexical-semantic relatedness processing during on-line sentence comprehension, and the potential similarity or differences between these two types of processes.

In the semantic network, a noun and a verb can be semantically related on the basis of shared features or/and co-occurrence in event and language such as *thief* and *arrest* [4]–[6]. Meanwhile, we can also analyze the thematic relation between the noun and the verb. Thematic relations are not equal to the lexical-semantic relatedness that is based on mere shared features or co-occurrence. For example, although both *arrest* and *pilfer* are semantically related to *thief*, *thief* bears an agent role in the action event defined by *pilfer* and a patient role in the action event defined by *arrest*; *fireworks* and *disturb* do not co-occur and do not share similar features, but *fireworks* bears an agent role in the context of someone *being disturbed* by the *fireworks*. In comparison to the lexical-semantic relatedness, thematic relations have their own characteristics. First, the thematic relation is a system of structural/causal relations in the semantic system, and the participants grouped by the thematic relation perform complementary roles: e.g., agent is a participant that the meaning of the verb specifies as doing or causing something; patient is a participant that the verb characterizes as being having something happen to it, and as being affected by what happens to it. Secondly, thematic relations group objects, concepts, or people together by virtue of their participation in the action event [4], [7], [8]. In short, in the semantic system, semantic relatedness can be considered as the inverse semantic distance between two lexically expressed concepts, and thematic relation is the structural/causal relation organized by action verbs. Although Jackendoff long ago had endorsed the position that thematic roles can be derivable from semantic representations [8], the exact relationship between thematic relation processing and semantic relatedness processing is still not clear. We don’t know whether, during on-line sentence comprehension, thematic role computing engages specialized systems as compared with the processing of lexical-semantic relatedness.

Some neurological studies have examined the difference between thematic relation and similarity-based semantic relation by using words pairs or pictures as materials. They found that thematic relation and similarity-based semantic relation are subserved by two functionally distinct systems [9]–[13]. For example, in an EEG experiment, Maguire and colleagues let healthy adults listen to thematically related (e.g., leash-dog), similarity-related (e.g., horse-dog), or unrelated (e.g., desk-dog) noun pairs and perform a lexical decision task. The results revealed that similarity-based semantic relation and thematic relation induced different oscillatory patterns. Specifically, the theta power increased over right frontal areas for thematic versus similarity-based relationships and the alpha power increased over parietal areas for similarity-based versus thematic relationships [12]. In a large-scale study, Schwartz and colleagues [13] examined picture naming errors produced by individuals with aphasia. They found that individuals differed in their tendency to produce similarity-based semantic errors (coordinate, superordinate, or subordinate noun substitutions) versus thematic errors (an object that co-occurred with the target in the context of an action or event). Moreover, a higher proportion of similarity-based errors is correlated with lesions affecting the left anterior temporal lobe (ATL) that is already well established as a critical hub for feature-based semantic category relations [14], [15]; a higher proportion of thematic errors is correlated with the left temporoparietal junction (TPJ) that has been established as a critical region for event-based and action-based relations [10], [16]. In short, evidences coming from neurological impairments and neuroimaging studies indicate that, during information processing, thematic relations engage important different neural processes as compared with the semantic relation based on shared features [12].

Recently, some EEG (electrophysiological) and fMRI studies also examined the process of thematic analysis in sentence context and its relationship with other aspect of meaning, such as world knowledge. For example, the EEG study conducted by Kuperberg and colleagues found that, in comparison with non-violated verbs (e.g. “…at breakfast the boys would eat…”), world knowledge violated verbs (e.g. “…at breakfast the boys would plant…”) evoked robust N400 effect and small P600 effect. Distinct from that evoked by world knowledge violated verbs, animacy thematically violated verbs (e.g. “…at breakfast the eggs would eat…”) evoked robust P600 effect but no N400 effect [17]. The following fMRI study further revealed that, relative to other sentence types, world knowledge violations led to increased activity within the left anterior inferior frontal cortex, reflecting participants’ increased efforts to retrieve semantic knowledge about the truth value of the sentence proposition in the real world; in contrast, animacy thematic violations engaged a frontal/inferior parietal/basal ganglia network considered to mediate the execution and comprehension of actions [18]. According to the authors, the above results reflect a distinction between the two types of knowledge: world knowledge and thematic relation.

Overall, the previous studies revealed that thematic relation processing is distinct from other aspects of semantic processing, such as the similarity-based semantic relation [12], [13] and world knowledge [17], [18]. However, the exact relationship between thematic role computing and other aspects of semantic processing during on-line sentence comprehension is still unclear due to the following reasons. First, even though some studies directly compared the difference between thematic relations and similarity-based semantic relations and made important contributions to our understanding of the semantic system, they focused on the relations between isolated concepts or nouns [9]–[13]. For example, they used isolated words or pictures as materials and let the subject to perform a kind of semantic priming task, in which the subject didn’t need to decide the agent or patient role of the nouns. The thematic relationship they examined was mainly the co-occurrence of one noun with another noun in the same action event. However, during real sentence comprehension, thematic role analysis must involve deciding the agent or patient role of the verb arguments. Therefore, the thematic analysis revealed by those semantic priming studies is not exact the same as the thematic role interpretation in the sentence context. Second, although some studies examined the process of thematic role analysis in the sentence context, they were interested in the differences between animacy-thematic processing and world knowledge processing. So, they didn’t directly compare the differences between thematic processing and lexical-semantic relatedness processing [17], [18]. Third, previous studies mainly focused on the differences between thematic processing and other aspects of semantic processing, it is still unclear how they interact with each other during on-line sentence comprehension. In short, we still don’t know whether thematic role computing is distinct from lexical-semantic relatedness processing during the on-line process of sentence comprehension, and how they interact with each other.

Therefore, the aim of the present study was to investigate the potential differences in the neural systems involved in thematic role computing and lexical-semantic relatedness processing during on-line sentence comprehension, and how these two types of process interact with each other.

Mandarin Chinese sentences were used to investigate these experimental questions. Different from some of the Indo-European languages, Mandarin Chinese doesn’t have case marking and subject-verb agreement; these phenomena, therefore, can’t help to assign the thematic role to an argument of Chinese sentence. Moreover, since word order in Mandarin Chinese is considerably free, the definition of thematic role can’t be just based on the structural position [19], [20]. Therefore, by using Mandarin Chinese sentences as materials, we could investigate the relationship between thematic role computing and semantic relatedness processing without the confounding effects coming from the above mentioned phenomena. In the present study, native speakers were asked to read Mandarin Chinese sentences for comprehension. The basic structure of those sentences was “Noun+Verb+‘le’+a two-character word”, with the Noun being the sentence-initial argument. The Verb disambiguates the Noun as an actor or undergoer of the event being described (agent vs. patient). Meanwhile, the Noun and the Verb were highly semantically related or lowly semantically related (high-relatedness vs. low-relatedness).

In the present study, a behavioral experiment was first conducted to examine the participants’ behavioral responses to the experimental sentences in the four experimental conditions. Then, the EEG technique was used to examine the experiment questions due to its high temporal resolution. Although thematic role conflict or violation has been found to elicit a P600 effect [17], semantic integration and thematic roles disambiguation has been shown to correlate with the N400 effect [20]–[23]. Therefore, the ERP component of interest in the present study is the N400 component. ERPs just reveal the phase-locked neural activities. Besides ERP, the oscillatory brain activities were also analyzed, which reveal both the phase-locked and non-phase-locked neural activities. Specific frequency bands presumably important for semantic processing, action processing and sentence-level syntactic processing were selected. These included theta (4–7 Hz), beta (15–30 Hz) and low-gamma (35–45 Hz) frequency bands. The theta power (4–7 Hz) increases have been found for semantic violation in language comprehension; and the theta power increases in temporal lobe are considered to be related to lexical-semantic retrieval operations [24]–[26]. The low gamma band power (35–45 Hz) has been reported to increase for semantically congruent words as compared with words incongruent with respect to their sentence context [26], [27]. It appears that normal sentence-level semantic unifications are accompanied by increases in the low gamma band synchronization. Moreover, sentence-level syntactic unification operations are associated with power variation in the beta frequency band (13–18 Hz) that is disrupted when the syntactic unification becomes problematic [28]. In addition, the neural synchronization around the beta band is also found to vary as a result of motor activity and mental motor simulation (e.g., during the generation or processing of action verbs) (15–25 Hz, 19–25 Hz, or 20–30 Hz) [29]–[31]. Those oscillatory brain activities can provide additional evidences for the underlying cognitive processes. In the present study, if thematic relation processing and semantic relatedness processing were mediated by the same neural systems, they would activate the same pattern of ERP results and brain oscillations results. Otherwise, thematic processing would activate a different pattern of ERP and oscillatory results as compared with semantic relatedness processing. Meanwhile, by examining the way of interaction between them, we could know the exact relationship between thematic and lexical-semantic processing during on-line sentence comprehension.

## Behavioral Experiment

### Method

#### Ethics statement

All participants provided written informed consent in accordance with the Declaration of Helsinki. The ethics committee of the Institute of Psychology, Chinese Academy of Sciences approved this study, its participant-recruitment procedure and its methodology.

#### Participants

Twenty university students (10 women; mean age = 21.5 years; SD = 1.9) participated in the experiment for cash. All participants were native speakers of Mandarin Chinese. All of them had normal or normal-to-corrected vision.

#### Stimuli

In the present study, 42 pairs of Mandarin Chinese sentences were constructed. As seen from Table 1, the basic structure of these sentences is “ Noun+Verb+‘le’+a two-character word”, with the Noun being a sentence-initial argument. The word ‘le’ is an auxiliary word, which indicates that the action demoted by the verb has already happened. The Nouns and the Verbs are all double –character words. Meanwhile, the verbs are all transitive verbs and the Nouns are always inanimate. The Noun in the sentences is ambiguous between the actor and undergoer of the action described by the Verb. On the one hand, we manipulated the semantic-relatedness between the Noun and the Verb: for 21 pairs of sentences, the Noun and the Verb in every sentence are highly semantically related; for the other 21 pairs of sentences, the Noun and the Verb in every sentence are lowly semantically related (high-relatedness vs. low-relatedness). On the one hand, we manipulated the thematic relationship between the Noun and the Verb. That is, every pair of sentences had the same Noun, but different Verbs: the Verb disambiguates the Noun as an actor or an undergoer of the event being described (agent vs. patient). Together, they realized a full factorial design with all combinations of Semantic Relatedness and Thematic Role (“high-relatedness, agent”, “high-relatedness, patient”, “low-relatedness, agent”, “low-relatedness, patient”) (see Table 1 and Text S1 for examples of experimental sentences).

**Table 1:** Example stimuli of all the four conditions used in the current study.

Conditions	Examples (Noun +Verb+‘le’+a two-character word)	
High-agent	棉被/温暖/了/孩子	Quilt/warm/‘le’/child
		The quit has warmed the child.
High-patient	棉被/缝制/了/几床	Quilt/sew/‘le’/several+classifier
		Several quilts have been sewn.
Low-agent	烟花/惊扰/了/婴儿	Fireworks/disturb/‘le’/baby
		The fireworks have disturbed the baby.
Low-patient	烟花/浸泡/了/几桶	Fireworks/soak/‘le’/several barrels
		Several barrels of fireworks have been soaked.

Note: High-agent indicates ‘high-relatedness, agent’ condition; High-patient indicates ‘high-relatedness, patient’ condition; Low-agent indicates ‘low-relatedness, agent’ condition; Low-patient indicates ‘low-relatedness, patient’ condition. The underlined words are the critical words (Verb).

In addition, we added a time adverbial or a place adverbial preceding the Noun in order to prevent the initial argument being at the beginning of the sentence. Those adverbial words were counterbalanced in the four experimental conditions.

In Mandarin Chinese, the basic word order is SVO (subject+verb+object, namely, agent+verb+patient). Subject-drop occurred frequently in Chinese. When the agent is not coded, at the requirement of information distribution, or rather, driven by Topic, the patient argument is moved to the sentence initial position, resulting in the “patient+verb+…” sentence [32]–[34]. Even though no passive morphology is used, the “Patient+Verb+ …” construction can indicate the passive relations semantically, since Chinese is a paratactic language, valuing the semantic coherence instead of the formal cohesion [35]. Moreover, in the present study, the two-character verbs are all verb-complement compound (e.g., 烧开 heat-boiling) or sequential-verb compound (e.g., 浸泡 soak) (These two kinds of verbs were counter-balanced between different conditions). The first character in the verb-complement compound is a verb and the second one is a complement describing the state caused by the verb. The two characters in the sequential-verb compound are all verbs and have similar meaning, with the former emphasizing the action and the latter emphasizing the result. That is, both kinds of compound verbs in the “Patient+Verb+‘le’+…” sentences emphasize the following two aspects: the action initiated by the implicit agent and the state of the patient caused by the action. Therefore, the characteristics of Mandarin Chinese as well as the verbs we used suggested that, in the present study, the initial argument in the “Patient+Verb+‘le’+…” sentence is the patient of the Verb.

**Figure 1:**
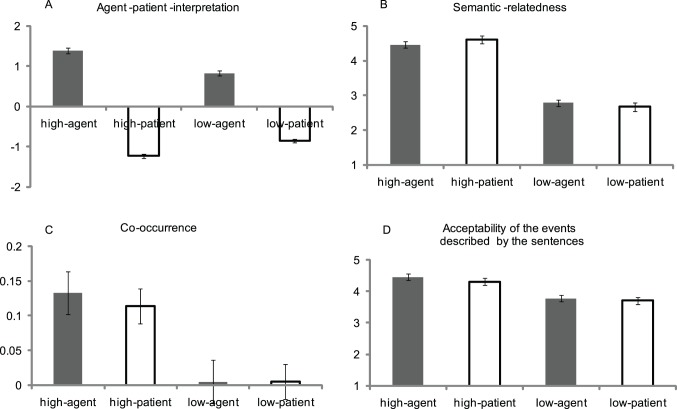
The results of Argument interpretation pre-test and Semantic relatedness pre-test. A (Argument interpretation pre-test): −2 indicated that the Noun was absolutely interpreted as an undergoer (Patient); 2 indicated that the Noun was absolutely interpreted as an actor (Agent). B (Semantic relatedness between the Noun and the Verb pre-test): The larger the number (from 1 to 5), the higher the level of the semantic relatedness is. C (Co-occurrence between the Noun and the Verb pre-test): the cloze probability of the Verb when the Noun was presented. D (Sentence event acceptability pre-test): The larger the number (from 1 to 5), the more acceptable the event described by the sentence is. High-agent indicates ‘high-relatedness, agent’ condition; High-patient indicates ‘high-relatedness, patient’ condition; Low-agent indicates ‘low-relatedness, agent’ condition; Low-patient indicates ‘low-relatedness, patient’ condition.

To confirm that the Noun was indeed interpreted as an actor or an undergoer, 16 participants who didn’t attend the behavioral and EEG experiments were presented with the Noun-Verb pairs and asked to mark the thematic role of the Noun on a 5-point scale (from −2 to 2). −2 indicated that the Noun was absolutely interpreted as an undergoer; 2 indicated that the Noun was absolutely interpreted as an actor. The ANOVAs with Semantic Relatedness (between item factor) and Thematic Role (within item factor) as independent factors revealed a significant main effect of Thematic Role (F_(1,40)_ = 1441.42, *p*<.0001). The rating score in the agent condition was larger than that in the patient condition for both the high-relatedness (F_(1,40)_ = 1069.39, *p*<.0001) and low-relatedness sentences (F_(1,40)_ = 440.60, *p*<.0001). In addition, the main effect of Semantic Relatedness didn’t reached significance (F_(1,40)_ = 2.51, *p* = .121) (see Figure 1). The results indicated that the manipulation of thematic relationship was successful.

To validate the degree of semantic relatedness between the Noun and the Verb, one the one hand, 16 subjects were asked to directly mark the semantic relatedness of the verb-noun pairs on a 5-point scale (from 1 to 5). The larger the number, the higher the degree of the semantic relatedness is. On the other hand, another 20 participants were presented with the Nouns and instructed to fill in the first verb that came to their mind (namely, to examine the co-occurrence between the Noun and the Verb). The ANOVAs with the semantic-relatedness score or the cloze probability score as dependent factors revealed that the score in the high-relatedness condition was larger than that in the low-relatedness condition both for the semantic-relatedness test (F_(1,40)_ = 237.22, *p*<.0001) and for the co-occurrence test (F_(1,40)_ = 20.61, *p*<.0001). The main effect of Thematic Role and the two-way Semantic Relatedness × Thematic Role interaction didn’t reach significance (all *p*s>.1) (see Figure 1). These results indicated that the manipulation of semantic relatedness was successful.

**Figure 2:**
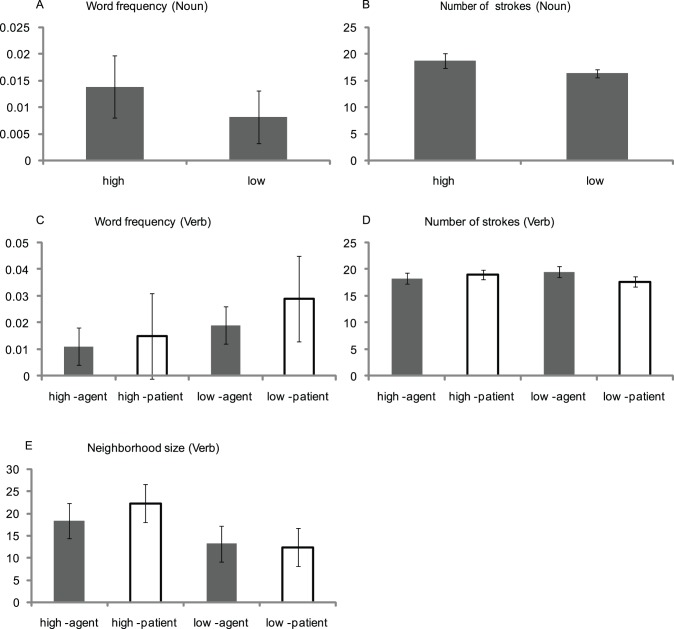
The word frequency and number of strokes of the Nouns. B: The word frequency, number of strokes, and neighborhood size of the Verbs. High-agent indicates ‘high-relatedness, agent’ condition; High-patient indicates ‘high-relatedness, patient’ condition; Low-agent indicates ‘low-relatedness, agent’ condition; Low-patient indicates ‘low-relatedness, patient’ condition.

We controlled the potential confounding factors, such as the word frequency and the number of strokes of the Nouns and the Verbs. On the one hand, for the Nouns, neither the word frequency (t _(40)_ = 0.82, *p* = 0.418) nor the number of strokes (t _(40)_ = 1.62, *p* = 0.115) showed significant difference between the high-relatedness and low-relatedness conditions. On the other hand, for both the word frequency, the number of strokes and the neighborhood size of the Verbs, the ANOVAs with Semantic Relatedness and Thematic Role as independent factors revealed neither significant main effects nor significant interaction (all *p*s >.1) (see Figure 2). Neighborhood size is the number of two-character words which share the initial syllable-word (the same consonant, vowel, and lexical tone) with the critical words. Overall, these results indicated that the word frequency, the number of strokes, and the neighborhood size were not confounding factors.

In addition, to confirm that the events described by the sentences in the agent condition were the same acceptable as those in the patient condition, another 24 participants were asked to mark the acceptability of the event described by every sentence on a 5-point scale (from 1 to 5). The larger the number, the more acceptable the sentence is. Previous studies found that a sentence whose initial argument bears an agent role is easier to process than other types of sentences, such as sentence with an initial patient argument [36]–[38]. To exclude that the difficulty of sentence processing influences the results of acceptability rating, all sentences were presented in their active version. For example, the sentence ‘棉被缝制了几床’(*quilt/sew/‘le’/several, Several quilts have been sewn*) coming from the patient condition was presented as ‘缝制了几床棉被’ (*sew/‘le’/several/quilt,* ‘S*omeone has sewn several quilts*), since subject-drop occurs frequently in Mandarin Chinese. That is, the sentence coming from the patient condition and its active version described exactly the same events. The ANOVAs with Semantic Relatedness and Thematic Role as independent factors resulted in a main effect of Semantic Relatedness (F_(1,40)_ = 44.03, *p*<.0001), suggesting that the events described by the high-relatedness sentences were more acceptable as compared with the low-relatedness sentences. It is reasonable that Semantic Relatedness influenced the acceptability of the sentence-event, since the Verbs and its Noun arguments had a higher level of co-occurrence and shared more similar features in the high-relatedness condition as compared with the low-relatedness condition. Importantly, the ANOVAs revealed that neither the main effect of Thematic Role (F_(1,40)_ = .82, *p* = .372) nor the two-way Semantic Relatedness× Thematic Role interaction (F_(1,40)_ = .114, *p* = .737) reached significance (see Figure 1). These results suggested that the acceptability of the events described by the sentences in the patient condition was the same acceptable as that in the agent condition.

The 42 pairs of experimental materials were grouped into 2 lists of 42 sentences according to the Latin square procedure based on the two experimental conditions (“agent” and “patient”). In each list, there were 10 (or 11) sentences for each of the four experimental conditions, and there was no repetition of the Noun and Verb across the four conditions. In addition, there were also 100 filler sentences in every list. 40 of the filler sentences have the same sentence structure as the experimental sentences, namely “adverb+inanimate Noun+Verb+“le”+a two-character word”. For half of the 40 filler sentences, the Verb disambiguates the Noun as an agent; for the other half of these sentences, the Verb disambiguates the Noun as a patient. The remaining 60 filler sentences are all subject-predicate sentences. Participants were divided into two groups, with each group read only one list of materials. That is, each participant was presented with 42 experimental sentences and additional 100 filler sentences.

#### Procedure

Self-paced reading paradigm was used in this experiment. The participants were asked to read each sentence for comprehension. They had to read each sentence in a word-by-word fashion. Reading times for every word were recorded. At the end of each sentence, the participants were asked to judge the correctness of a question sentence regarding the meaning of the sentence just read. The question sentences (e.g., The clothes were dyed, Someone has sewn the quilts) followed the experimental sentences all had the correct meaning. The question sentences followed the filler sentences that have the same sentence structure as the experimental sentences all had the wrong meaning. For the remaining 60 filler sentences, half of the question sentences were correct and half of them were wrong. The key press for ‘correct’ and ‘wrong’ was counterbalanced between the participants. After a short practice session that consisted of 10 sentences, all of the experimental sentences and filler sentences were presented in one block of approximately 20 minutes.

#### Data analysis

For all participants, the accuracy rate was equal to or higher than 95% (The accuracy rate of two participants is equal to 95%). The reading times of the disambiguating Verb, the reading time of the auxiliary word ‘le’ (namely, the word immediately following the Verb), and the accuracy of the question sentence were analyzed respectively. For each kind of reading times, we deleted the data outside 3 standard deviations and further deleted the data followed by incorrect response. Then the remaining data were subjected to analyses of variance (ANOVA) with participants (F1) and items (F2) as random factors respectively. The independent factors were Semantic Relatedness (high-relatedness vs. low-relatedness) and Thematic Role (agent vs. patient). For participant analysis, Semantic Relatedness and Thematic Role were both within-participant factors; for item analysis, Thematic Role was within-item factor and Semantic Relatedness between-item factor.

### Results

#### Accuracy rate of the question sentence

**Figure 3:**
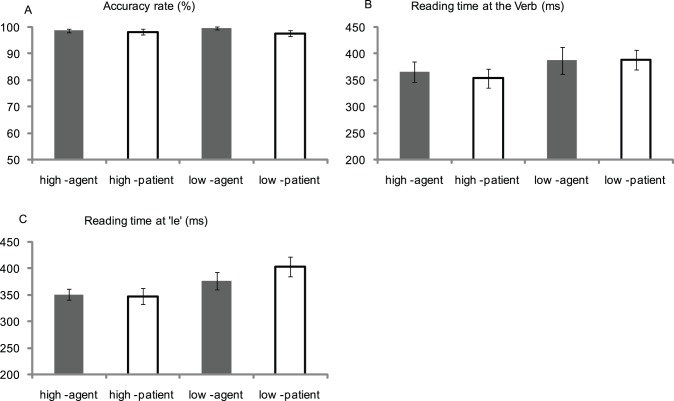
The results of the behavioral experiment. A: Accuracy rate for the question sentences in the four experimental conditions; B: The reading time at the disambiguating Verb; C: The reading time at the word ‘le’ immediately following the Verb. High-agent indicates ‘high-relatedness, agent’ condition; High-patient indicates ‘high-relatedness, patient’ condition; Low-agent indicates ‘low-relatedness, agent’ condition; Low-patient indicates ‘low-relatedness, patient’ condition.

For the accuracy rate of the question sentence, neither the participant analysis nor the item analysis found significant main effects or interaction (all ps >.1, see Figure 3).

#### Reading time at the verb

For the reading time at the disambiguating Verb (see Figure 3), the ANOVAs resulted in a significant main effect of Semantic Relatedness (F_1_(1,19) = 13.08, p<0.005, MSE = 1210.22; F_2_(1,40) = 3.90, p = 0.055, MSE = 4232.63), indicating that the reading time in the low-relatedness condition was longer than that in the high-relatedness condition.

#### Reading time at ‘le’

For the reading time at the word immediately following the Verb (see Figure 3), the ANOVAs still revealed a significant main effect of Semantic Relatedness (F_1_(1,19) = 18.29, p<0.0001, MSE = 1824.58; F_2_(1,40) = 16.37, p<0.0001, MSE = 2306.85), indicating that the reading time in the low-relatedness condition was longer than that in the high-relatedness condition. Importantly, the participant analysis revealed a two-way Semantic Relatedness × Thematic Role interaction (F_1_(1,19) = 8.14, p<0.01, MSE = 558.72; F_2_(1,40) = 2.44, p = 0.126, MSE = 2021.08). Further simple analysis showed that, the Semantic Relatedness effect reached significance both in the agent condition (F_1_(1,19) = 5.71, p<0.05, MSE = 1163.71; F_2_(1,40) = 3.78, p = 0.059, MSE = 2336.19) and in the patient condition (F_1_(1,19) = 25.65, p<0.0001, MSE = 1219.59; F_2_(1,40) = 15.26, p<0.0001, MSE = 2291.73). Another way of simple analysis revealed that when the Noun and the Verb were lowly semantically related, the reading time in the patient condition was longer than that in the agent condition (F_1_(1,19) = 10.28, p<0.005, MSE = 689.95; F_2_(1,40) = 4.15, p<0.05, MSE = 2021.08); in contrast, when the Noun and the Verb were highly semantically related, there was no significant difference between the agent and patient conditions (F_1_(1,19) = .21, p = 0.655, MSE = 601.56; F_2_(1,40) = .03, p = .865, MSE = 2021.08).

In summary, the results of the behavioral experiment confirmed that the experimental sentences can be correctly understood by the participants. Moreover, this behavioral study revealed that the low semantic-relatedness sentences were harder to process than the high semantic-relatedness sentences both in the agent and patient conditions; however, the patient condition induced longer reading time than the agent condition only when the verb and its noun argument were lowly semantically related. Although this behavioral study gave us some preliminary results on the relationship between lexical-semantic processing and thematic processing, it required participants to perform an additional task during sentence comprehension, such as pressing key after every word and answering a question after every experimental sentence. In contrast, the EEG technique can explore the process of sentence comprehension without additional tasks and can provide more parameters (both ERPs and brain oscillatory activity) reflecting the underlying mechanisms. Therefore, the EEG experiment was conducted to further examine the relationship between thematic role computing and lexical-semantic relatedness processing, and to examine the potential differences in the neural systems involved in these two types of process.

## EEG Experiment

### Method

#### Ethics statement

All participants provided written informed consent in accordance with the Declaration of Helsinki. The ethics committee of the Institute of Psychology, Chinese Academy of Sciences approved this study, its participant-recruitment procedure and its methodology.

#### Participants

Twenty university students (12 women; mean age = 22.0 years; SD = 2.5) participated in the experiment for cash. All participants were native speakers of Mandarin Chinese. All of them had normal or normal-to-corrected vision, and were right-handed and neurologically healthy.

#### Stimuli

The experimental sentences (42 pairs of Mandarin Chinese sentences) in the EEG experiment were exactly the same as those used in the behavioral experiment.

To guarantee that there were enough number of trials to obtain the reliable ERP components, the participants read all of the experimental sentences (21 sentences for each of the four experimental conditions), and every experimental sentence was presented twice. In addition, there were also 100 filler sentences in every list. For the filler sentences, many kinds of sentence structure were used in order to prevent the participants detecting the aims of the experiment: 14 sentences with “Agent+Verb+‘le’+Patient” structure and 14 sentences with “Patient+Verb+‘le’+…”, which include different number of characters as compared with the experimental sentences; 32 sentences with “Subject+adjective phrase” or “Subject+‘shi’ (is/are)+adjective phrase”, with the adjective phrase describing the properties of the subject; 20 sentence with “NP1+ ‘ba’+NP2+ verb +…” structure (*ba* indicates the preceding argument is agent); 20 sentence with “NP1+ ‘bei’+NP2+ verb +…” structure (*bei* indicates the preceding argument is patient). The whole list of sentences (268 sentences) was divided into four blocks, with the first and the second presentations of the same experimental sentence being separated by one block.

#### Procedure

After the electrodes were positioned, participants were asked to read each sentence for comprehension. Meanwhile, their EEG signals were recorded. Each trial started with a fixation “+” (duration: 1000 ms) in the center of the screen. After the fixation, the sentence was presented word by word (e.g., the adverb//Noun//Verb//‘le’//the two-character word), with each word appearing for 300 ms, with an inter stimulus interval (ISI) of 300 ms. In order to present the ERP effect on the Nouns spilling over into the Verbs, we add a 200 ms jitter (ISI varying randomly from 200 to 400 ms) to the inter stimulus interval between the Noun and the Verb. To ensure that the participants indeed read the sentences for comprehension, at the end of each of the 80 filler sentences in all of the materials, they were asked to judge the correctness of a question sentence regarding the meaning of the sentence just read. After a short practice session consisting of ten sentences, the trials were presented in four blocks of about thirty minutes totally.

#### EEG acquisition

EEG was recorded (0.05–100 Hz, sampling rate 500 Hz) from 64 Ag/AgCl electrodes mounted in an elastic cap, with an on-line reference linked to the left mastoid and off-line algebraic re-reference linked to the left and right mastoids. EEG and EOG data were amplified with AC amplifiers (**Synamps**, Neuroscan Inc.). Vertical eye movements were monitored via a supra- to sub-orbital bipolar montage. A right-to-left canthal bipolar montage was used to monitor horizontal eye movements. All electrode impedance levels (EEG and EOG) were kept below 5 kΩ.

#### ERP analysis

The data of one subject, whose accuracy rate for the question sentence judgment was lower than 85%, was deleted from further analysis. The accuracy rates of the remaining subjects were all higher than 95%. Another three participants were further rejected from the final analysis due to EEG-artifacts, experimenter error or technical problems with recording. Thus, 16 participants remained for subsequent analysis. For ERP analysis, the raw EEG data were first corrected for eye-blink and filtered with a band-pass filter 0.1–40 Hz. Then, the filtered data were divided into epochs ranging from 100 ms before the onset of the critical words to 1000 ms after the onset of the critical words. A time window of 100 ms preceding the onset of the critical words was used for baseline correction. Trials contaminated by eye movements, muscle artifacts, electrode drifting, amplifier saturation, or other artifacts were identified with a semiautomatic artifact rejection (automatic criterion: signal amplitude exceeding ±75 uV, followed by a manual check). Trials containing the abovementioned artifacts were rejected (14.3% overall). Rejected trials were evenly distributed among conditions. Finally, averages were computed for each participant, each condition, and at each electrode site before grand averages were calculated across all participants.

**Figure 4:**
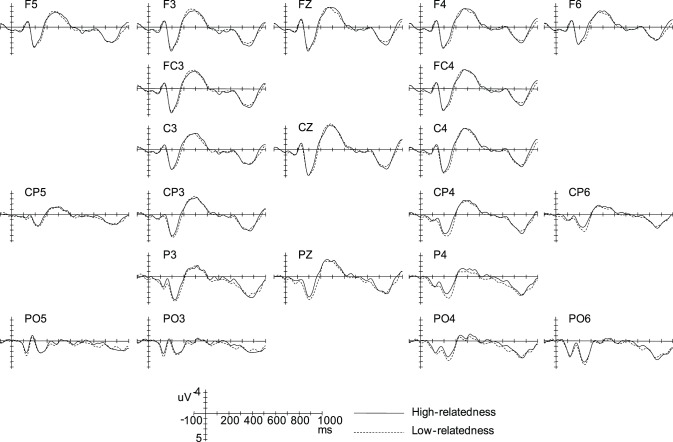
Grand-average ERPs time-locked to the Nouns in the low-relatedness and the high-relatedness conditions.

Figure 4 showed overlays of the ERP waveforms time-locked to the Nouns (sentence-initial argument) in the high- and low-relatedness conditions. It looks that there is no obvious difference in the ERP waveforms elicited by the two conditions.

**Figure 5:**
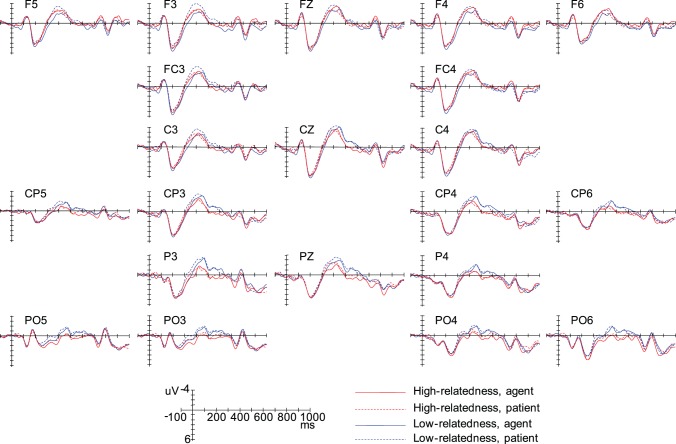
Grand-average ERPs time-locked to the Verbs in the four experimental conditions.

**Figure 6:**
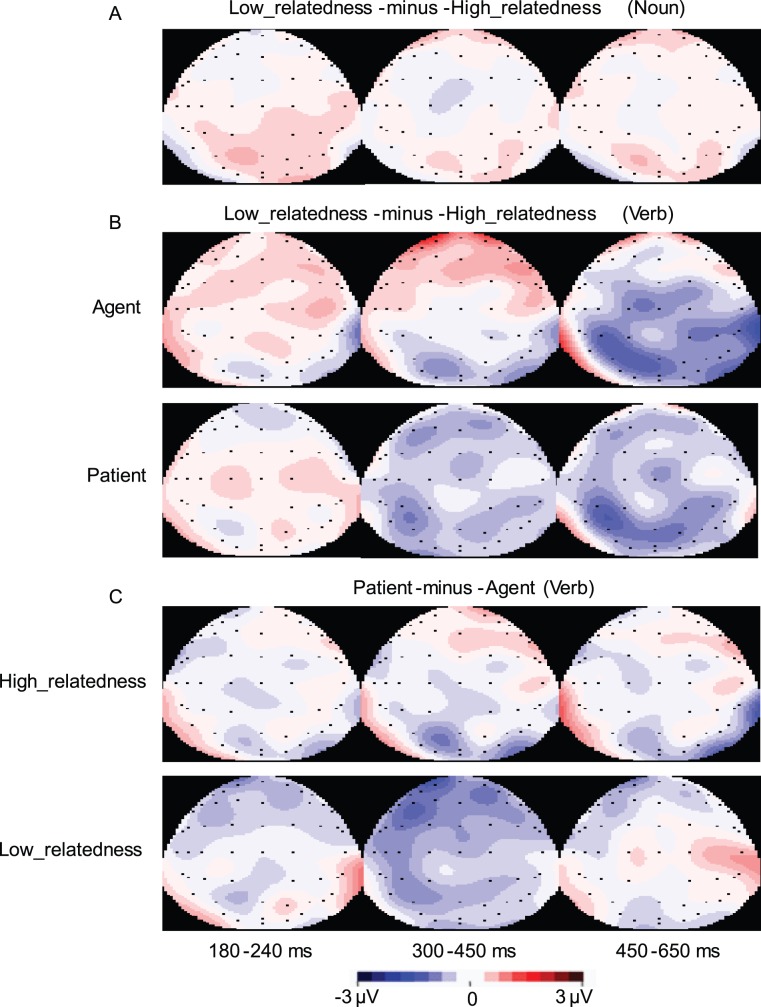
Topographies of the corresponding ERP effects. A: Topography of the semantic relatedness effects (time-locked to the Noun). B: Topography of the semantic relatedness effects (time-locked to the Verb) at different levels of thematic relation. C: Topography of the thematic relation effects (time-locked to the Verb) at different levels of semantic relatedness.

Figure 5 showed overlays of the ERP waveforms time-locked to the Verbs in the four experimental conditions. Relative to the high-relatedness condition, the low-relatedness condition elicited a larger negative deflection, which peaked around 430 ms after the onset of the Verbs and had a parietal distribution (see Figure 6). We classified this negativity effect as a N400 effect, since its latency and topography fit the standard characteristics of an N400 effect. In addition, relative to the agent condition, the patient condition elicited a larger negative deflection, which peaked around 400 ms and had a frontal-central distribution. We also classified this negativity effect as a N400 effect, since previous studies had observed the same frontally distributed N400 effect [39], [40].

For the ERPs time-locked to the Verbs, statistical analyses were done on the mean amplitude in the 180–240 ms (for P2), 300–450 ms (for N400), and 450–650 ms (the residual effect of N400) latency ranges following the onset of the Verbs. Analyses of variance were conducted on a selection of midline electrodes and lateral electrodes respectively. For the midline electrode sites (Fz, Cz, and Pz), the mean amplitude values were entered into ANOVAs with Semantic Relatedness (high-relatedness vs. low-relatedness), Thematic Role (agent vs. patient), and Anteriority (frontal, central, vs. parietal) as independent factors. For lateral electrodes, the mean amplitude values were entered into ANOVAs with Hemisphere (left vs. right) as an additional factor and lateral electrodes (F5/F3/FC3; F4/F6/FC4; C3/CP5/CP3; C4/CP4/CP6; P3/PO5/PO3; P4/PO4/PO6) nested under Hemisphere.

For the ERPs time-locked to the Nouns, statistical analyses were done on the mean amplitude in the same time windows (180–240 ms, 300–450 ms, and 450–650 ms latency ranges) following the onset of the Nouns. The ANOVAs at the position of Noun were the same as those at the position of Verb except that the independent factors didn’t include Thematic Role. When the degree of freedom in the numerator was larger than one, the Greenhouse-Geisser correction was applied.

#### EEG time-frequency analysis

Event-related spectral perturbation (ERSP) was applied to characterize the oscillatory brain activities. ERSP represented the time-frequency representations (TFRs) averaged across single trials, which contain both phase-locked and non-phase-locked brain activities. The raw EEG data were screened off-line for eye movements, muscle artifacts, electrode drifting, and amplifier blocking in a critical window that ranged from 1000 ms before to 1600 ms after the onset of the Verbs. Subsequently, ERSP were calculated using EEGLAB 10.2.5.5b (http://www.sccn.ucsd.edu/eeglab) running under Matlab 7.5 (MathWorks, Natick, MA, USA). We applied Morlet wavelet decomposition [41] to the 2600-ms data epoch. We used 200 linearly-spaced time points and a series of 48 log-spaced frequencies ranging from 3 Hz to 50 Hz, with 1.5 cycles at the lowest frequency and 12.25 cycles at the highest frequency. Power values were normalized with respect to a −600 to −200 ms pre-stimulus baseline and transformed into decibel scale (10*log10 of the signal), yielding the ERSP.

Based on previous studies on language processing [24], [26], [30] and the present brain oscillations results, statistic analysis was performed on the theta (4–7 Hz), beta (15–30 Hz), and low-gamma (35–45 Hz) band frequency bands. The ANOVAs in the consecutive 50-ms time windows and the permutation test implemented in the statcond function of EEGLAB toolbox was used to find out the time windows that the ERSP values were significantly distinguished. And then, the ERSP values for each subject over each electrode under each condition were averaged in the corresponding frequency-bands and time windows. The ERSP values were averaged for the left frontal (F7, F3, FC5, FC3), right frontal (F4, F8, FC4, FC6), left central (T7, C3, TP7, CP5), right central (C4, T8, CP6, TP8), left posterior (P7, P3, PO5, PO3), and right posterior (P4, P8, PO4, PO6) regions. Then, the data were analyzed by repeated-measures ANOVA with Semantic Relatedness, Thematic Role, Anteriority (frontal, central, and parietal), and Hemisphere (left, right) as independent factors.

### Results

#### ERP results at the noun

In all of the window latencies (180–240 ms, 300–450 ms, and 450–650 ms window latencies), the ANOVAs revealed neither significant main effects nor significant interactions (see Figure 4 and Figure 6).

#### ERP results at the verb

The ANOVA for the 180–240 ms window latency revealed neither significant main effects nor significant interactions (all ps>.1).

As seen from Figure 5 and Figure 6, in the 300–450 ms window latency, the ANOVAs revealed a significant main effect of Thematic Role, suggesting that the patient condition elicited a larger N400 than the agent condition (*F_midline_*(1,15) = 4.28, *p* = .056, *MSE* = 3.78; *F_lateral_*(1,15) = 8.94, *p*<.01, *MSE* = 3.73) (effect magnitude: −0.58 µV and −0.59 for midline and lateral analysis respectively). The ANOVAs also showed a significant two-way Semantic Relatedness × Anteriority interaction over the lateral electrodes (*F_lateral_*(2,30) = 9.65, *p*<.001, *MSE* = .81), due to the fact that the low- semantic condition evoked a larger N400 than the high-relatedness condition only over the parietal electrodes (*F_lateral_*(1,15) = 16.08, *p*<.005, *MSE* = 1.28; effect magnitude: −0.80 µV). Importantly, the three way Semantic Relatedness × Thematic Role × Anteriority interaction also reached significance (*F_lateral_*(2,30) = 7.25, *p*<.01, *MSE* = .64). Simple-simple analysis found that there was a significant two-way Semantic Relatedness × Anteriority interaction over the frontal electrodes (*F_lateral_*(1,15) = 5.51, *p*<.05, *MSE* = 3.00), but not over the central and parietal electrodes (all *p*s >.1); moreover, over the frontal electrodes, the patient condition elicited a larger N400 than the agent condition when the Verb and the Noun were lowly semantically related (*F_lateral_*(1,15) = 8.00, *p*<.05, *MSE* = 3.24), but not when they were highly semantically related (*F_lateral_*(1,15) = .21, *p* = .654, *MSE* = 2.10).

To explore the planned simple-simple effect of Semantic Relatedness (or Thematic Role) at each level of Thematic Role (or Semantic Relatedness) over the frontal, central, and posterior electrodes respectively, further simple-simple analysis was performed in the 300–450 ms window latency. On the one hand, it was found that, relative to the high-relatedness condition, the low-relatedness conditions elicited an enhanced N400 over the parietal electrodes, but not over the central and frontal electrodes both when the initial argument was disambiguated as an agent (*F_lateral_*(1,15) = 3.42, *p* = .083, *MSE* = 2.95; *F_lateral_*(1,15) = 0.09, *p* = .774, *MSE* = 4.84; *F_lateral_*(1,15) = 5.62, *p*<.05, *MSE* = 1.62 for frontal, central, and parietal electrodes respectively) and when it was disambiguated as a patient (*F_lateral_*(1,15) = 2.22, *p* = .157, *MSE* = 2.94; *F_lateral_*(1,15) = 3.64, *p* = .076, *MSE* = 2.58; *F_lateral_*(1,15) = 5.85, *p*<.05, *MSE* = 1.96 for frontal, central, and parietal electrodes respectively). On the other hand, relative to the agent condition, the patient condition evoked a larger N400 over the frontal, central, and parietal electrodes when the Noun and the Verb were lowly semantically related, which reached maximum over the frontal electrodes (*F_lateral_*(1,15) = 8.00, *p*<.05, *MSE* = 3.24; *F_lateral_*(1,15) = 4.45, *p* = .05, *MSE* = 2.75; and *F_lateral_*(1,15) = 6.26, *p*<.05, *MSE* = 1.18 for frontal, central, and parietal electrodes respectively); however, when the Noun and the Verb were highly semantically related, there was no significant difference in the ERPs elicited by the agent and patient conditions (*F_lateral_*(1,15) = .21, *p* = .654, *MSE* = 2.10; *F_lateral_*(1,15) = .36, *p* = .556, *MSE* = 3.41; and *F_lateral_*(1,15) = 2.51, *p* = .134, *MSE* = 2.19 for frontal, central, and parietal electrodes respectively).

The ANOVA for the 450–650 ms window latency resulted in a significant main effect of Semantic Relatedness, indicating that the low-relatedness condition elicited a larger negative deflection than the high-relatedness condition (*F_midline_*(1,15) = 6.97, *p*<.05, *MSE* = 5.12; *F_lateral_*(1,15) = 7.91, *p*<.05, *MSE* = 6.64) (effect magnitude: −0.87 µV and −0.64 for midline and lateral analysis respectively). This Semantic Relatedness effect was qualified by a two-way Semantic Relatedness × Anteriority interaction (*F_midline_*(2,30) = 4.66, *p*<.05, *MSE* = 1.66; *F_lateral_*(2,30) = 9.00, *p*<.005, *MSE* = 1.31). Further simple analysis revealed that the Semantic Relatedness effect reached significance over the parietal electrodes (*F_midline_*(1,15) = 12.33, *p*<.005, *MSE* = 2.35; *F_lateral_*(1,15) = 15.20, *p*<.001, *MSE* = 2.47) and central electrodes (*F_midline_*(1,15) = 6.58, *p*<.05, *MSE* = 2.41; *F_lateral_*(1,15) = 9.37, *p*<.01, *MSE* = 3.07), but not over the frontal electrodes(*F_midline_*(1,15) = .38, *p* = .547, *MSE* = 2.53; *F_lateral_*(1,15) = .32, *p* = .578, *MSE* = 2.86).

In the present study, every experimental sentence was present twice for every participant. In order to examine whether the repetition of experimental sentences harmed the experimental results, further split-half analysis was conducted with Presentation Order (first vs. second) as an additional independent factor. The split-half analysis demonstrated that there were no significant interactions with Presentation Order with regard to the effects of interest (or, when there were, they were not due to effects only arising in the first or the second half). These results indicated that the experimental results were not harmed by the repetition of experimental sentences.

Overall, the thematic relation variation evoked a broadly distributed N400 effect, which reached maximum over the frontal electrodes; the Semantic Relatedness variation elicited a central-posterior N400 effect, which reached maximum over the parietal electrodes. The N400 effect elicited by the thematic relation variation was modulated by the semantic relatedness between the Verb and its argument; however, the posterior N400 effect evoked by the semantic relatedness variation was in some degree independent from the thematic relation between the Verb and the Noun.

#### TFRs results at the verb

For each of the three frequency bands (4–7 Hz, 15–30 Hz, and 35–45 Hz), we conducted ANOVAs with mean power as dependent factor in consecutive 50-ms time windows from 0 to 1000 ms after the onset of the verb. Significance or marginal significance (for the same effect) on 3 consecutive bins was taken as evidence for the existence of a certain effect. According to this criterion, the simple main effect of Thematic Role over the left hemisphere was significant or marginally significant from 300 to 600 ms in the beta band (15–30 Hz); the simple main effect of Semantic Relatedness over left-posterior electrodes was significant from 450 to 700 ms in the theta band (4–7 Hz). According to the topographies of the above effects, electrode P3 was selected to further examine the Thematic Role effect (agent-first vs. patient-first) or Semantic Relatedness effect (inanimate vs. animate) by using the permutation test in the EEGLAB software package. The permutation test showed the similar pattern of results as the consecutive 50-ms ANOVAs. Then, based on these results, the ERSP values for each participant over each electrode under each condition were averaged in the beta band (15–30 Hz) within the latency range of 300–600 ms and in the theta band (4–7 Hz) within the latency range of 450–700 ms. In addition, in the low-gamma (35–45 Hz) frequency band, the consecutive 50-ms ANOVAs and the permutation test didn’t find reliably significant effect in all of the window latencies from 0 to 1000 ms after the onset of the Verb.

**Figure 7:**
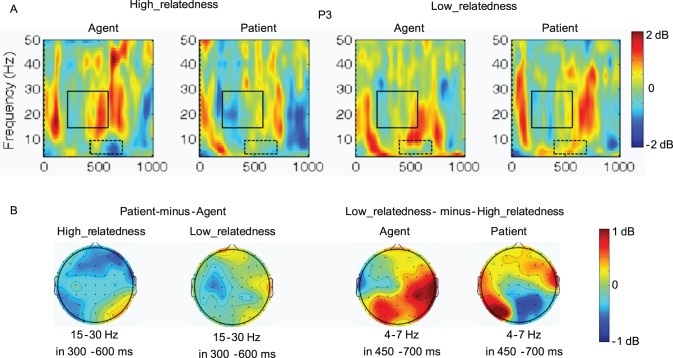
Time-frequency analysis of electroencephalogram series in the four experimental conditions. A: Event-related spectral perturbation (ERSP) from electrode P3. Black square frames indicate the time window and frequency for which there are significant differences between the agent and patient conditions; dotted black square frames indicate the time window and frequency for which there are significant differences between the high_relatedness and low_relatedness conditions. B: Topographies of the thematic relation effect of beta power (15–30 Hz) and the semantic relatedness effect of theta power (4–7 Hz).

As seen from Figure 7, in the 450–700 ms theta (4–7 Hz) window, the ANOVA resulted in a significant three-way Semantic Relatedness ×Anteriority × Hemisphere interaction (*F* (2,30) = 6.36, *p<*.01, *MSE* = 9.55). Further simple-simple analysis showed that the low-relatedness condition induced significantly larger theta power increases than the high-relatedness condition over the left parietal electrodes (*F* (1,15) = 4.85, *p<*.05, *MSE* = 30.08; effect magnitude: 3.02 dB).

In the 300–600 ms high-beta (15–30 Hz) window, the ANOVA revealed a significant two-way Thematic Role × Hemisphere interaction (*F* (1,15) = 4.88, *p<*.05, *MSE* = 8.37) due to the fact that the patient condition induced larger beta power decreases than the agent condition over the left hemisphere (*F* (1,15) = 7.34, *p<*.01, *MSE* = 8.82, effect magnitude: −1.16 dB) but not over the right hemisphere (*F* (1,15) = .07, *p = *.800, *MSE* = 14.90, effect magnitude:0.14 dB).

## Discussion

The present study examined the potential differences between thematic role computing and lexical-semantic relatedness processing during on-line sentence comprehension, and the interaction between these two types of processes. The major results were that, thematic relation variations evoked a frontal N400 effect, which appeared only when the initial argument and the disambiguating verb were lowly semantically related; however, lexical-semantic relatedness variations elicited a posterior N400 effect regardless of the thematic role of the initial argument. In addition, the brain oscillations results showed that, relative to the agent condition, the patient condition induced larger power decreases in the beta band (15–30 Hz in 300–600 ms); in contrast, relative to the high-relatedness condition, the low-relatedness condition induced larger power increases in the theta band (4–7 Hz in 450–700 ms). These results are discussed below in more detail.

### The Interaction between Thematic Role Computing and Semantic Relatedness Processing during On-line Sentence Comprehension

The results of the present study revealed that, relative to the agent condition (the verb disambiguating the initial argument as an actor), the patient condition (the verb disambiguating the initial argument as an undergoer) elicited a larger N400 only when the verb and the initial argument were lowly semantically related; however, when the two words were highly semantically related, there was no difference in the ERPs elicited by the agent and the patient conditions. Previous studies have suggested that verb meanings are represented in structured situation (which could be realized as an event-schema), and thematic roles and argument structure are quickly accessed by the processor when the verb is recognized [42]–[46]. In the present study, it might be that, when the initial argument and the verb were highly semantically related, the thematic role of the sentence-initial arguments could be immediately retrieved from the event-schema stored in memory, hence both the agent and the patient roles being easily assigned and no differences in the ERPs elicited by them. However, when the initial argument and the verb were lowly semantically related, the agent and the patient roles could not be directly accessed from the event-schema stored in memory, and had to be computed on-line based on the semantic features of the verb and the argument. Meanwhile, the thematic relation is a causal/structural relationship and the first participant in an event function is preferred interpreted as the actor [7], [47]. Therefore, in the low-relatedness condition, it was more difficult to process when the verb disambiguated the initial argument as a patient as compared with when the verb disambiguated the initial argument as an agent, hence the patient condition eliciting an enhanced N400. This N400 effect evoked by the thematic role variation was in line with the ‘actor-first preference’ effect (namely, the tendency to analyze an initial ambiguous argument as the actor of the event described by the verb) found by early studies [20], [36], [37], [38]. As to the cognitive mechanisms underlying the Thematic Role effect observed in the present study, some researchers assumed that the sentence with an initial agent argument appears more frequently in real-time language than the sentence with an initial patient role [48], hence the former being easier to process. However, based on available cross-linguistic facts, other researchers claimed that the actor-first preference is attributable to an epiphenomenon of minimal events, which leads to an initial ambiguous argument being interpreted as the sole argument in the sentence (thereby, sole argument+intransitive verb) [20], [36], [38]. The reanalysis from the sole argument reading to an agent-first reading is less costly than the revision to a patient-first reading, hence the sentence with an initial agent argument being easier to process. That is, different accounts have been put forward to explain the Thematic Role effect observed in the present study, which needs to be explored further in the future. Anyway, the present results suggested that, during on-line sentence comprehension, thematic role assignment is modulated by the lexical-semantic relatedness between the verb and its arguments.

In contrast, semantic relatedness variations elicited a posterior N400 effect both when the initial argument was an agent and when it was a patient. Specifically, in the window latency of 300–450 ms after the onset of the verb, although there was a significant interaction between Semantic Relatedness and Thematic Role, further analysis found that the low-relatedness condition evoked an enhanced N400 regardless of the thematic role of the initial argument. In the following time window (450–650 ms), only the effect of Semantic Relatedness reached significance, which was not modulated by the thematic role of the initial argument. In addition, the brain oscillations results also revealed that, relative to the high-relatedness condition, the low-relatedness condition induced larger theta band (4–7 Hz in 450–700 ms after the onset of the verb) power increases regardless of the thematic role of the initial argument. Those results suggested that, during on-line sentence comprehension, semantic relatedness processing is in some degree independent from the thematic relation between the verb and its arguments.

Previous studies have already begun to investigate the relationship between thematic relation and other aspects of semantic information. The present study is interesting because it is the first time to examine the interaction between thematic role computing and semantic relatedness processing during on-line sentence comprehension. Our results shed new light on this field by showing that, in the sentence context, thematic role computing is modulated by the lexical-semantic relatedness between the verb and its arguments; however, lexical-semantic relatedness processing is somewhat independent from the thematic relations.

As mentioned in the introduction, different from some of the Indo-European languages, Mandarin Chinese has neither case marking nor subject-verb agreement, hence these phenomena not helping to assign the thematic role of an argument. Under these circumstances, the sentence context and the semantic relationship between the different sentence constituents might play a more important role in thematic processing of Mandarin Chinese sentences as compared with other languages. However, it’s less likely that the modulating effect of lexical-semantic relatedness on thematic processing observed the present study is language specific due to the following reasons. First, although the specific linguistic cues used to comprehend sentence might sometimes be different between Mandarin Chinese and other languages, the comprehension of Mandarin Chinese sentence was found to depend on some universal strategies, such as syntactic prediction [49], [50]. For example, Chen and Yan found that, during the comprehension of Mandarin Chinese sentence, the reader can use the word *huozhe* (used as “or” in English) at the beginning of a sentence to predicatively construct the syntactic structure [50]. Second, although some morphosyntactic cues in some kinds of language (such as case marker) can help to determine the thematic role of the noun arguments, to understand the whole sentence, these thematic roles need to be linked to the lexical representations of the verb. That linking process might be influenced by the lexical-semantic relatedness between the verb and its arguments. Moreover, the thematic relation between the verb and the noun arguments is in fact derivable from semantic representation [8]. Therefore, we think that the interaction between thematic role computing and lexical-semantic relatedness processing observed in the present study might be universal across various languages, which needs to be further explored with other languages as materials.

### The Distinction of Thematic Role Computing from Semantic Relatedness Processing

Another important aim of the present study was to investigate the potential differences between thematic role computing and semantic relatedness processing during on-line sentence comprehension. The results showed that although both thematic role variations and semantic-relatedness variations elicited N400 effects, the N400 effect elicited by the former was broadly distributed and reached maximum over the frontal electrodes, and the N400 effect elicited by the latter had a posterior distribution. We argued that, the different topography of the N400 effects suggested that thematic role computing and semantic relatedness processing might engage distinct neural processes during on-line sentence comprehension.

The brain oscillations results also provided evidences for the distinction of thematic processing from lexical-semantic relatedness processing during on-line sentence comprehension. The brain oscillations results revealed that, relative to the high-relatedness condition, the low-relatedness condition induced larger theta band (4–7 Hz in 450–700 ms) power increases in the left-posterior electrodes. As mentioned earlier, the theta power increases have been found for semantic violation in language comprehension; and the theta power increases around temporal lobe are considered to be related to lexical-semantic retrieval operations [24]–[26]. In the present study, it might be that, relative to the high-relatedness condition, the verbs in the low-relatedness condition were more difficult to retrieve from the memory and harder to integrate into the sentence context, hence inducing theta power increases. Different from semantic relatedness processing, thematic role computing in the present study induced power variations around the beta frequency bands. That is, relative to the patient condition, the agent condition induced larger beta band (15–30 Hz in 300–600 ms) power decreases at the left hemisphere regardless of the semantic-relatedness between the verb and the initial argument. The variation of beta band power has been found to related to sentence-level syntactic unification (around 13–18 Hz) [28] and has been repeatedly found during action verb processing (e.g., verb generation) and preparation of an item-related motor reaction (around 15–25 Hz, 19–25 Hz, or 20–30 Hz) [29], [30], [31], [51]. In the present study, the sentences in the patient condition had different sentence structure as compared with those in the agent condition. Meanwhile, thematic relation is organized by action verbs and its understanding is about deciding who is doing what to whom in an action event. Therefore, in the present study, thematic role computing during sentence comprehension induced brain oscillatory variations in the frequency bands related to sentence-level syntactic unification and, more importantly, in the frequency bands related to action verb processing. The engagement of action-based system to thematic processing is consistent with previous studies. For example, in a picture naming study, Schwartz and colleagues found that individuals made a high portion of thematic errors usually had lesions affecting the left temporoparietal junction (TPJ) that has been established as a critical region for event-based and action-based relations [13]. Kuperberg and colleagues also found that, in comparison with non-violated verbs (e.g. “…at breakfast the boys would eat…”), animacy–thematically violated verbs (e.g. “…at breakfast the eggs would eat…”) led to increased activity within a frontal/inferior parietal/basal ganglia network known to mediate the execution and comprehension of goal-directed action [18]. Overall, the results of the present study revealed that, semantic relatedness processing induced power variations in the theta band (4–7 Hz) that has been associated with semantic retrieval and semantic integration processes; in contrast, thematic role computing induced power variations around the beta band (15–30 Hz) that is known to mediate sentence-level syntactic processing and action-based comprehension. In accordance with the ERP results, the brain oscillations results also indicated that, during on-line sentence comprehension, thematic role computing and lexical-semantic relatedness processing might be underlined by functionally distinct neural basis.

As mentioned in the introduction, previous studies have already found that thematic relation processing engaged distinct neural systems as compared with other aspects of semantic processing. Those studies made important contributions to our understanding of the semantic system and language comprehension [25], [26], [12], [13]. However, some of the previous studies focused on the relationship between isolated nouns. The cognitive process revealed by the semantic priming task they used is not equal to the thematic role assignment process in the real sentence context [12], [13]. Although some studies investigated the process of thematic role assignment in sentence context, they were interested in the differences between thematic role violation (namely, thematic role reversal) and world knowledge violation [25], [26]. The present study is the first study investigating the relationship between thematic role computing and lexical-semantic relation processing during on-line sentence comprehension. The present results were consistent with the previous studies by revealing that thematic relation processing is different from other aspects of meaning processing. More importantly, the present results also provided new insights by showing that, during on-line sentence comprehension, the neural basis underlying thematic role computing is distinct from that underlying lexical-semantic relatedness processing. Our results suggested that the thematic relation and its processing are not redundantly specified in the semantic system.

## Conclusions

In conclusion, the present study investigated the relationship between thematic role computing and lexical-semantic relatedness processing during on-line sentence comprehension. Our results demonstrated that, during sentence comprehension, thematic role computing is modulated by the semantic relatedness between the verb and its argument; in contrast, lexical-semantic relatedness processing is in some degree independent from the thematic relations. More importantly, the present results indicated that, during on-line sentence comprehension, thematic role computing and lexical-semantic relatedness processing are mediated by distinct neural systems. Our results have important implications for both theories of sentence processing and of semantic representation. It is suggested that, although thematic roles can be derivable from semantic representations, thematic relation and its processing is not redundantly defined in the semantic system.

## Supporting Information

Text S1 **Parts of the experimental sentences (not including the adverbial word at the beginning of the sentence).**
(DOCX)
