# Supplementary material for: The Relation between Thematic Role Computing and Semantic Relatedness Processing during On-Line Sentence Comprehension
Source: PLoS One. 2014 Apr 22;9(4):e95834. doi: 10.1371/journal.pone.0095834 (PMC3995949; doi:10.1371/journal.pone.0095834)
Supplement: Text S1 — Parts of the experimental sentences (not including the adverbial word at the beginning of the sentence). (DOCX) [file pone.0095834.s001.docx]

Text S1: Parts of the experimental sentences (not including the adverbial word at the beginning of the sentence)

**High-semantic:**

1. Agent 热水/烫伤/了/手腕 Hot-water/scald/‘le’/wrist

The Hot-water has scalded the wrist.

Patient 热水/烧开/了/几壶 Hot-water/boil/‘le’/several kettles

Several kettles of hot-water have been boiled.

1. Agent 颜料/漂染/了/衣裳 Dyestuff/dye/‘le’/clothes

The dyestuff has dyed the clothes.

Patient 颜料/挑选/了/几盒 Dyestuff/select/‘le’/several boxes

Several boxes of dyestuff have been selected.

1. Agent 露水/打湿/了头巾 Dew/wet/‘le’/scarf

The dew has wetted the scarf.

Patient 露水/采集/了/一些 Dew/collect/‘le’/some

Some dew has been collected.

1. Agent 冰箱/冷冻/了/牛肉 Refrigerator/freeze/‘le’/beef

The refrigerator has frozen the beef.

Patient 冰箱/修理/了/几台 Refrigerator/fix/‘le’/several + classifier

Several refrigerators have been fixed.

1. Agent 汗水/浸湿/了/衣衫 Sweat/drench/‘le’/coat

The sweat has drenched the coat.

Patient 汗水/抹去/了/一些 Sweat/wipe/‘le’/some

Some sweat has been wiped

1. Agent 棉被/温暖/了/孩子 Quilt/warm/‘le’/child

The quit has warmed the child.

Patient 棉被/缝制/了/几床 Quilt/sew/‘le’/several + classifier

Several quilts have been sewn.

1. Agent 纸巾/吸干/了/污水 Tissue paper/suck up/‘le’/slops

The tissue paper has sucked up the slops.

Patient 纸巾/撕碎/了/几张 Tissue paper/tear up/‘le’/several pieces + classifier

Several pieces of tissue paper have been torn up.

1. Agent 雨水/滋润/了/树苗 Rainwater/moisten/‘le’/saplings

The rainwater has moistened the saplings.

Patient 雨水/储蓄/了/一些 Rainwater/save/‘le’/some

Some rainwater has been saved.

**Low-semantic:**

1. Agent 古玩/引来/了/商人 Antique/attract…to come here/‘le’/businessman

The antiques have attracted the businessman to come here.

Patient 古玩/背来/了/几件 Antique/bring here by carrying on the back /‘le’/several + classifier

Several antiques have been brought here by carrying on the back.

1. Agent 烟花/惊扰/了/婴儿 Fireworks/disturb/‘le’/baby

The fireworks have disturbed the baby.

Patient 烟花/浸泡/了/几桶 Fireworks/soak/‘le’/several barrels

Several barrels of fireworks have been soaked.

1. Agent 家书/诱降/了/将军 Letter from home/lure into surrender /‘le’/general

The letter from home has lured the general into surrender.

Patient 家书/抄袭/了/几份 Letter from home/copy/‘le’/several + classifier

Several letters from home have been copied.

1. Agent 泪水/愚弄/了/大众 Tear/fool/‘le’/the people

The tears have fooled the people.

Patient 泪水/酝酿/了/一些 Tear/brew/‘le’/some

Some tears have been brewed.

1. Agent 生姜/辣坏/了/孩子 Ginger/burn/‘le’/child

The ginger has burned the child.

Patient 生姜/撒入/了/几片 Ginger/add/‘le’/several pieces

Several pieces of ginger have been added.

1. Agent 电视/揭露/了/真相 Television/ferret out/‘le’/truth

The television has ferreted out the truth.

Patient 电视/拉来/了/一台 Television/bring here/‘le’/one + classifier

One television has been brought here.

1. Agent 导弹/撼动/了/大地 Missile/shake/‘le’/ground

The missile has shaken the ground.

Patient 导弹/托运/了/几枚 Missile/consign for shipment/‘le’/several + classifier

Several missiles have been consigned for shipment.

1. Agent 写真/欺骗/了/评委 Photographs/deceive/‘le’/judge

The photographs have deceived the judge.

Patient 写真/拆除/了/几本 Photographs/dismantle/‘le’/several books

Several books of photographs have been dismantled.
